# Untapped potential of multicenter studies: a review of cardiovascular risk prediction models revealed inappropriate analyses and wide variation in reporting

**DOI:** 10.1186/s41512-019-0046-9

**Published:** 2019-02-22

**Authors:** L. Wynants, D. M. Kent, D. Timmerman, C. M. Lundquist, B. Van Calster

**Affiliations:** 1 Department of Development and Regeneration, KU Leuven, Herestraat 49, box 7003, 3000 Leuven, Belgium; 2 Predictive Analytics and Comparative Effectiveness (PACE) Center, Institute for Clinical Research and Health Policy Studies, Tufts Medical Center, 800 Washington St, Box 63, Boston, MA 02111 USA; 3 Department of Biomedical Data Sciences, Leiden University Medical Center, PO Box 9600, Leiden, 2300RC The Netherlands; 4 Department of Obstetrics and Gynecology, University Hospitals Leuven, Herestraat 49, 3000 Leuven, Belgium; 5 Department of Epidemiology, CAPHRI Care and Public Health Research Institute, Maastricht University, PO Box 9600, 6200 MD Maastricht, The Netherlands

**Keywords:** Clinical prediction model, Multicenter, Cardiovascular disease

## Abstract

**Background:**

Clinical prediction models are often constructed using multicenter databases. Such a data structure poses additional challenges for statistical analysis (clustered data) but offers opportunities for model generalizability to a broad range of centers. The purpose of this study was to describe properties, analysis, and reporting of multicenter studies in the Tufts PACE Clinical Prediction Model Registry and to illustrate consequences of common design and analyses choices.

**Methods:**

Fifty randomly selected studies that are included in the Tufts registry as multicenter and published after 2000 underwent full-text screening. Simulated examples illustrate some key concepts relevant to multicenter prediction research.

**Results:**

Multicenter studies differed widely in the number of participating centers (range 2 to 5473). Thirty-nine of 50 studies ignored the multicenter nature of data in the statistical analysis. In the others, clustering was resolved by developing the model on only one center, using mixed effects or stratified regression, or by using center-level characteristics as predictors. Twenty-three of 50 studies did not describe the clinical settings or type of centers from which data was obtained. Four of 50 studies discussed neither generalizability nor external validity of the developed model.

**Conclusions:**

Regression methods and validation strategies tailored to multicenter studies are underutilized. Reporting on generalizability and potential external validity of the model lacks transparency. Hence, multicenter prediction research has untapped potential.

**Registration:**

This review was not registered.

**Electronic supplementary material:**

The online version of this article (10.1186/s41512-019-0046-9) contains supplementary material, which is available to authorized users.

## Introduction

Clinical predictive models (CPMs) are clinically useable mathematical equations that relate multiple predictors for a particular individual to the probability of risk for the presence (diagnosis) or future occurrence (prognosis) of a particular outcome [1]. They are an increasingly common and important methodological tool for patient-centered outcomes research and for clinical care. By providing evidence-based estimates of the patient’s probability of health outcomes, CPMs enable clinicians and patients to make decisions that are more rational and consistent with a patient’s own risks, values, and preferences.

It is no surprise that many researchers use multicenter datasets as a substrate to develop clinical prediction models. To be useful, clinical prediction models must be reliable in new patients, potentially including patients from different hospitals, countries, or care settings. Recruitment at multiple sites makes it easier to collect sufficient data to estimate model parameters reliably, especially when the outcome of interest is a rare event.

Currently, the TRIPOD guidelines do not mention any requirements that are specific to multicenter studies, other than reporting the number and location of centers [1, 2]. Nonetheless, multicenter studies pose particular challenges to statistical data analysis and offer new opportunities. On the one hand, a key assumption underlying common regression techniques is violated. Observations are not independent as they are clustered within hospitals; patients within a hospital may be more alike than patients from different hospitals. On the other hand, the fact that hospital populations differ from one another may shed light on the generalizability of the model.

Studies have shown that mixed (i.e., random intercept) and fixed (i.e., center variables) effects regression allow to study differences between centers in event rates and predictor effects and may even provide better predictions [3–8]. Leave-center-out cross-validation has been proposed to efficiently assess the generalizability of the model to centers not included in the development set [9, 10]. For example, small center effects and successful cross-validation of a model developed in multiple tertiary care centers in one country strongly indicate generalizability to other tertiary centers in that country. However, transportability of the model to clinical care settings or distinct populations not represented in the data can never be guaranteed [11–13]. In the example above, predictive performance in primary or secondary care or in foreign centers (“transportability”) remains to be assessed in external validation studies.

Hence, multicenter studies are extremely interesting if they are representative of the settings in which the model is intended to be used. This is uncertain if centers that participate in studies differ from those who do not, for example, because they have an academic interest or specialization in the disease under study (selection bias) [14]. Cases identified from specialist centers may not be representative of all cases in the general population, and patients without the condition may have been referred there because they presented with many risk factors, distorting regression estimates (referral bias). Moreover, the predictive performance of a model may differ between subgroups in a population (spectrum bias), which has led to the recommendation to use the prevalence in the studied setting as a guide when evaluating whether the reported predictive performance is applicable to a particular clinical setting [15–18].

The purpose of this research is to investigate the properties, analysis, and reporting of multicenter studies in the Tufts PACE Clinical Prediction Model Registry, a comprehensive database of clinical prediction models for cardiovascular disease [19, 20]. In addition, we provide simulated illustrations of consequences of common design and analysis choices.

## Methods

We searched the Tufts PACE Clinical Prediction Model Registry for multicenter studies. This registry contains published clinical prediction models for patients at risk for and with known cardiovascular disease. The inclusion and exclusion criteria and electronic search strategy are published in detail elsewhere [19, 20]. Briefly, the registry was constructed from a PubMed search for English-language articles containing newly developed prediction models published from January 1990 to March 2015. It includes prognostic and diagnostic models to predict binary outcomes (e.g., myocardial infarction or death). Only articles that show the model in a format that allows readers to make individual predictions were included (e.g., an equation, a point score, an online calculator).

We considered studies labeled in the registry as multicenter and published between January 2000 and March 2015. From these, a randomly selected subset of 50 papers underwent full-text screening. When a paper presented multiple prediction models, we selected the primary model as identified by the authors. Where no primary model was identified, we selected the one with the smallest number of events per variable (EPV). We extracted sample size and other dataset characteristics for the development dataset. In two studies, the development dataset was single center (due to a geographical train-test split of multicenter data). In these cases, we described the complete multicenter dataset.

One researcher (LW) extracted the following and entered it in an excel spreadsheet:

- The total sample size of the development dataset;
- The number of events of interest in the development dataset;
- The ratio of the number of patients with events divided by the total sample size (ignoring censoring in time-to-event data);
- The number of centers;
- The ratio of the sample size in the largest and smallest center;
- Center sizes, or any descriptive information related to center size;
- A description of the setting in which the model was developed (e.g., all tertiary/academic centers, a community cohort, mixed settings);
- The type of study data used for model development (registry/cohort, trial, individual patient data meta-analysis (IPD-MA));
- The regression technique used for model development;
- Whether an external validation was included in the study (a random train-test split is not considered external validation);
- How many external validation datasets were used;
- Whether external validation happened in the same center (or community) as model development;
- Whether external validation happened in the same country as model development;
- Whether external validation happened in the same care setting as model development;
- Whether external validation used data from the same time period as model development;
- Statements regarding the generalizability of the prediction model (including selection, referral, and spectrum bias).

When a publication did not report on these items, but the authors referred to earlier publications or a study website when describing the methodology, we screened these sources. The retrieved information was cross-checked with recorded information in the Tufts PACE database, and conflicts were resolved [19, 20]. We summarized all extracted data as frequencies, medians, ranges, and interquartile ranges.

We did not register this review, nor did we publish a review protocol. We used the PRISMA checklist to prepare this manuscript [21].

To highlight potential issues with common design and analysis choices for multicenter prediction models, we supplemented the review with illustrations, the detailed methods of which are provided in the Appendix. Briefly, in the first illustration, we compare marginal predicted probabilities (obtained by using standard logistic regression ignoring clustering) to conditional predicted probabilities from a mixed effects model. We simulated a dataset with a binary outcome with event rate 0.33 and a single standard normal predictor (with the same distribution across centers) with an effect of 0.8 on the logit scale. We generated data for 500 centers, with 1000 observations each, and let the intercepts vary per center with a standard deviation of 1 or 0.5. We fitted a standard logistic regression model and a mixed effects logistic regression model and evaluated predictions in terms of discrimination and calibration.

In the second illustration, we exemplify non-transportability with a published example of a risk score to predict recurrent cardiovascular events [22] and by using a real multicenter clinical dataset that was collected to develop preoperative prediction models for ovarian cancer diagnosis [23]. The dataset contains information on 3439 patients from 12 oncology referral centers and 1664 patients from 8 general hospitals. We consider a binary outcome *Y* (ovarian malignancy) and two predictors: age and the log-transformed CA125 biomarker value. The observed relations (from logistic regression) between the outcome and predictors in tertiary care and secondary care are taken to be the true models in each setting. Using resampling techniques, we generated a typical multicenter model development dataset in tertiary care, a large external validation set in tertiary care, and a large external validation set in secondary care. We made calibration plots and computed C-statistics to assess the generalizability and transportability of the model.

## Results

**Fig. 1:**
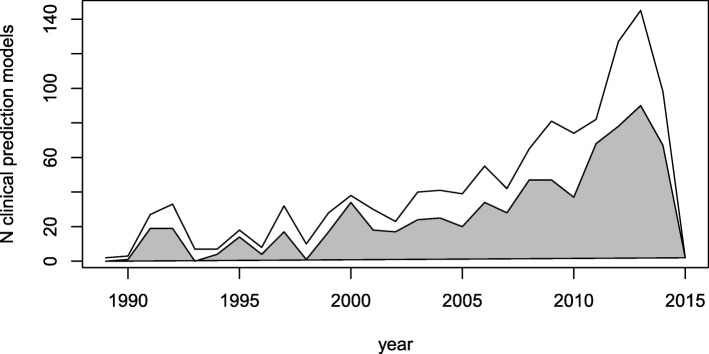
Evolution of the yearly total number of prediction models (upper line) and the yearly number of prediction models built with multicenter data (shaded area) in the Tufts PACE Clinical Prediction Model Registry

The number of clinical prediction models published per year showed a strong increase over time. The proportion of models built using multicenter data increased slightly over time. Sixty-four percent (602/944 models) of models published after 2000 used multicenter data (see Fig. 1).

**Fig. 2:**
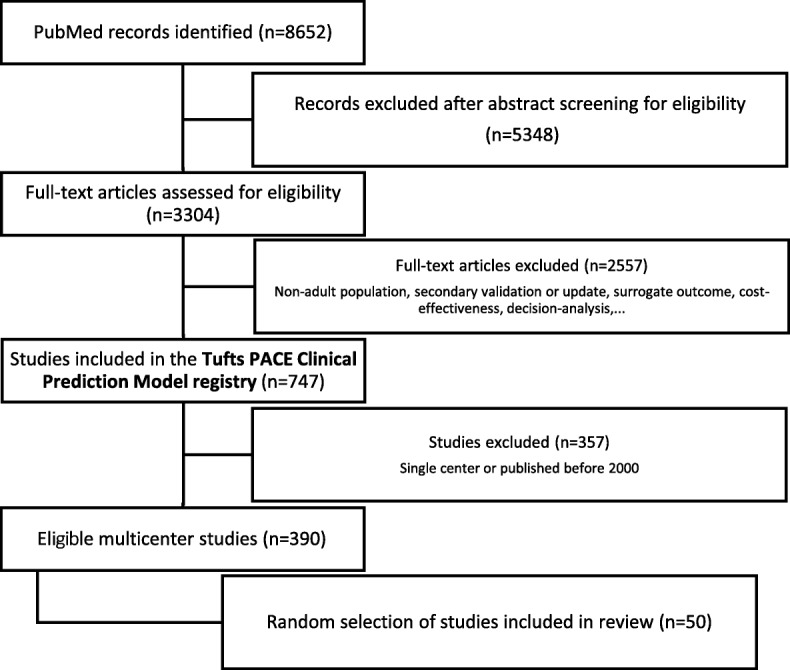
The Tufts PACE Clinical Prediction Model Registry was searched for relevant multicenter articles published after 2000

Of all studies included in the Tufts PACE Clinical Prediction Model Registry, 52% (390/747 studies) were multicenter studies published after 2000 (see flowchart in Fig. 2). We discuss the details of a random subset of 50 studies (Table 1) [24–73]. Forty of them analyzed observational multicenter data. Six created a prediction model making secondary use of multicenter trial data. For one study, the reporting on the data source was equivocal. Three studies were individual patient data meta-analyses (IPD-MA), of which two combined datasets from multiple community cohorts and one combined data from multiple trials (some of them are multicenter trials themselves). In IPD-MA of existing study data, the primary source of heterogeneity is at the study level and not at the center level. Hence, from these three studies, study-level information was extracted and analyzed instead of center-level information (e.g., number of studies in the IDP-MA, *n* in each of the original studies, statistical account of clustering within studies).

**Table 1:** Key characteristics of 50 selected publications

1st author	Journal	Year	Data type	*N* development	*N* events development	No. of centers	Regression technique	Clinical setting	International	Externally validated	Generalizability statement
Agarwal	J Heart Lung Transplant	2012	Registry/cohort	339	111	3	Cox ignoring clustering	All tertiary/academic	No	Yes	Generalizability uncertain: high-risk population (but validated in low-risk population)
Alsonso	J Am Heart Assoc	2013	IPD-MA	18,556	1186	3	Stratified Cox	Community/population cohort	No	Yes	Generalizability uncertain: selection/referral/spectrum bias
Amin	J Am Heart Assoc	2013	Trial	19,121	988	674	Cox ignoring clustering		Yes	Yes	Generalizability uncertain: selection/referral/spectrum bias
Bekelis	J Neurointerv Surg	2014	Registry/cohort	4551	6	4573	Logistic ignoring clustering	Mix of community hospitals, diverse w.r.t. academic status	No	No	Validation needed
Carluccio	Eur J Heart Fail	2013	Registry/cohort	747	224		Cox ignoring clustering			No	Validation needed
Chung-Esaki	Am J Emerg Med	2014	Registry/cohort	249	18	2	Logistic ignoring clustering	All community hospitals		No	Validation needed
Cuschieri	J Dig Dis	2014	Registry/cohort	3218	107	10	Cox ignoring clustering	All veteran affairs hospitals	No	No	Generalizability uncertain: selection/referral/spectrum bias
de la Cámara	Cardiol J	2012	Registry/cohort	600	98	3	Logistic ignoring clustering	All tertiary/academic	No	No	
de Man-van Ginkel	Stroke	2013	Registry/cohort	382	54	3	Logistic ignoring clustering		No	No	Validation needed
den Exter	Chest	2013	Registry/cohort	1048	225	288	Logistic ignoring clustering	Mix of teaching and non-teaching hospitals	Yes	Yes	Claim generalizability of model
Dodson	J Am Coll Cardiol	2014	Registry/cohort	168,442	3032	1428	Mixed effects logistic		No	No	Generalizability uncertain: selection/referral/spectrum bias
Eichinger	J Am Heart Assoc	2014	Registry/cohort	553	150	4	Fine and Gray ignoring clustering		No	No	Validation needed
Enajat	Plast Reconstr Surg	2013	Registry/cohort	430	17	2	Logistic ignoring clustering	All tertiary/academic	No	No	Validation needed
Farooq	Eur Heart J	2012	IPD-MA	6309	175	7	Stratified logistic		Yes	No	Validation needed
Felker	J Card Fail	2004	Trial	949	90	78	Cox ignoring clustering	Mix of academic and community hospitals	No	No	Validation needed
Goff	J Am Coll Cardiol	2014	IPD-MA	24,626	2689	4	Cox ignoring clustering	Community/population cohort	No	No	Validation needed
Grant	Br J Surg	2013	Registry/cohort	11,423	312	140	Logistic ignoring clustering		No	No	Validation needed
Gupta	J Vasc Surg	2012	Registry/cohort	9556	170		Logistic ignoring clustering	Mix of academic and community hospitals	No	No	Generalizability uncertain: selection/referral/spectrum bias
Hannan	JACC Cardiovasc Interv	2013	Registry/cohort	54,223	558	58	Logistic ignoring clustering	Community/population cohort	No	Yes	Validation needed
Hubert	J Am Coll Cardiol	2013	Registry/cohort	565	48	2	Fine and Gray ignoring clustering	All tertiary/academic	No	No	Generalizability uncertain: selection/referral/spectrum bias
Iannuzzi	J Vasc Surg	2013	Registry/cohort	562,791	2862		Logistic ignoring clustering		No	No	Generalizability uncertain: selection/referral/spectrum bias
Iida	Eur J Vasc Endovasc Surg	2012	Registry/cohort	1058	497	11	Cox ignoring clustering		No	No	
Iung	Heart	2014	Registry/cohort	2552	253	34	Mixed effects logistic		No	No	Generalizability uncertain: high-risk population
Kay	J Am Coll Cardiol	2013	Registry/cohort	6037	169	3	Cox and fixed center effect			No	Generalizability uncertain: selection/referral/spectrum bias
Kramer	Heart Rhythm	2012	Registry/cohort	905	125	3	Logistic ignoring clustering	All tertiary/academic	No	No	Generalizability uncertain: selection/referral/spectrum bias
Ky	Circ Heart Fail	2012	Registry/cohort	1513	317	3	Cox ignoring clustering	All tertiary/academic	No	No	Validation needed
Liu	CNS Neurosci	2014		575	76	30	Logistic ignoring clustering	All tertiary/academic	No	No	Generalizability uncertain: selection/referral/spectrum bias
Myint	Int J Stroke	2014	Registry/cohort	12,355	2425	3	Logistic ignoring clustering	All tertiary/academic	No	No	Validation needed
Palmerini	JACC Cardiovasc Interv	2012	Trial	1692	173		Cox ignoring clustering		No	No	Validation needed
Pannucci	Chest	2014	Registry/cohort	6768	95	10	Logistic ignoring clustering		No	No	Validation needed
Park	Am J Cardiol	2012	Registry/cohort	63,118	217	126	Cox ignoring clustering		Yes	No	Claim generalizability of model
Perry	Stroke	2014	Registry/cohort	3906	86	8	Logistic ignoring clustering	All tertiary/academic	No	No	Validation needed
Piccini	Circulation	2013	Trial	14,155	575	1100	Cox and fixed region effect		Yes	Yes	Generalizability uncertain: selection/referral/spectrum bias
Pros	Eur J Vasc Endovasc Surg	2013	Registry/cohort	640	123	3	Reduce to single-center development set, then logistic	All tertiary/academic	No	Yes	Generalizability uncertain: high-risk population
Rao	JACC Cardiovasc Interv	2013	Registry/cohort	834,696	48,412	1142	Logistic ignoring clustering	Mix of teaching and non-teaching hospitals	No	No	
Rizzi	BMC Infect Dis	2014	Registry/cohort	1456	499	25	Logistic ignoring clustering	Mix of secondary and tertiary	No	No	Generalizability uncertain: high-risk population
Schellinger	Stroke	2013	Trial	192		68	Logistic ignoring clustering		Yes	No	
Singer	J Am Heart Assoc	2013	Registry/cohort	32,590	952	2	Reduce to single-center development set, then Cox	All community hospitals	No	Yes	Validation needed
Stiell	Acad Emerg Med	2013	Registry/cohort	559	65	6	Logistic ignoring clustering	All tertiary/academic	No	No	Validation needed
Takagi	J Am Coll Cardiol	2013	Registry/cohort	1286	76	47	Cox ignoring clustering		No	No	Validation needed
Than	Emerg Med Australas	2014	Registry/cohort	1974	305	2	Logistic ignoring clustering		Yes	Yes	Validation needed
Tolenaar	Circulation	2014	Registry/cohort	1034	110	12	Logistic ignoring clustering	All tertiary/academic	Yes	No	Validation needed
Trujillo-Santons	Am J Med	2015	Registry/cohort	15,280	173	125	Logistic ignoring clustering		Yes	No	Validation needed
Van De Bruaene	Int J Cardiol	2013	Registry/cohort	155	39	10	Cox ignoring clustering	All tertiary/academic	No	No	Validation needed
Van Hattum	Thromb Haemost	2012	Trial	482	287	6	Cox ignoring clustering		No	No	Validation needed
Wang	Cardiology	2013	Registry/cohort	1615	343	3	Logistic ignoring clustering		No	Yes	Validation needed
Wilson	Am J Med	2012	Registry/cohort	33,419	2394	5473	Cox and fixed region effect	Mix of public and private hospitals	Yes	No	Validation needed
Wimmer	Stroke	2012	Registry/cohort	10,186	177	364	Logistic and fixed effect for physician experience	Mix of academic and private hospitals	Yes	No	Generalizability uncertain: use of specific devices
Wimmer	J Am Heart Assoc	2013	Registry/cohort	14,387	366	5	Mixed effects logistic		No	No	Generalizability uncertain: high-risk population
Zheng	Front Med	2013	Registry/cohort	8602	215	43	Logistic ignoring clustering		No	No	Validation needed

Blank cells indicate unreported information

The sample size for model development in the 50 selected studies varied from 155 to 834,696 with a median of 2263 (IQR 667 to 13,705). Sixteen studies had between 100 and 1000 observations, 18 studies had between 1000 and 10,000 observations, 13 studies had between 10,000 and 100,000 observations, and 3 studies had more than 100,000 observations.

### Multicenter: a catch-all term

**Fig. 3:**
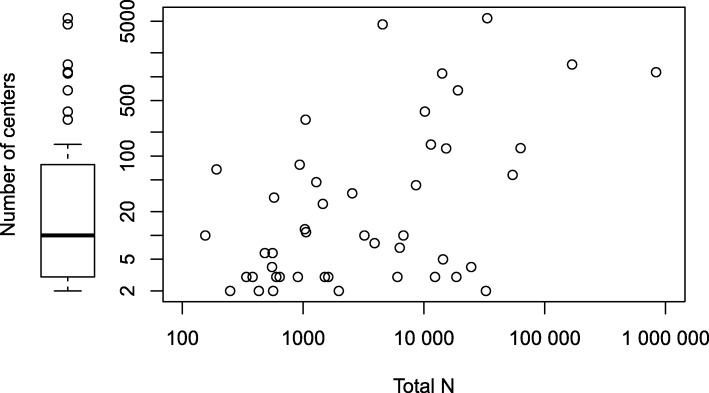
Total sample size and number of centers (logarithmic axis scales)

The number of centers contributing data to a multicenter study varied widely from just two centers to 5473 centers, with a median of 10 (IQR 3 to 76) (Fig. 3). As such, “multicenter” is a term covering anything from two researchers joining forces to large international registries. There was a moderate positive correlation between the total sample size and the number of contributing centers (Fig. 3; Spearman *ρ* 0.4). Four studies did not report the number of participating centers. Two of them were large studies (*N* = 562,791 and 9556) using data from a quality of care improvement program mentioning more than a hundred centers participated, one was a secondary analysis of a multicenter trial (*N* = 1692), and one was a cohort study (*N* = 747).

The median number of patients per center was 133 (range 1 to 16,295; IQR 62 to 530), but imbalance in center contributions was very common. Sample sizes for each included center were reported in only 9/50 studies. The ratio of largest to smallest center size could be computed in 10 studies. Among these, the median sample size ratio of the largest center to the smallest was 4 (range 1 to 34, IQR 3 to 7). Twelve additional studies contained summary statements on sample sizes (e.g., interquartile ranges) or hospital volume (e.g., number of beds, surgical volume, catchment population sizes). These indicated imbalance in center sizes in nine studies, were equivocal in one study, and, interestingly, described active control to limit imbalance in two studies. These active controls were including only the first 10 to 20 consecutive patients in each center and setting an upper inclusion limit for centers. The majority (28/50) did not report on center size imbalance. These tended to be smaller studies (median *N* 1654 versus 4294).

Multicenter collaborations were not exclusively used to study rare outcomes. The median ratio of the number of events to the total sample size was 0.07 (range 0.001 to 0.60, IQR 0.03 to 0.16, not reported in 1), and the median number of events 177 (range 6 to 48,412; IQR 98 to 497; not reported in 1).

### Clustered data is commonly ignored during analysis

**Fig. 4:**
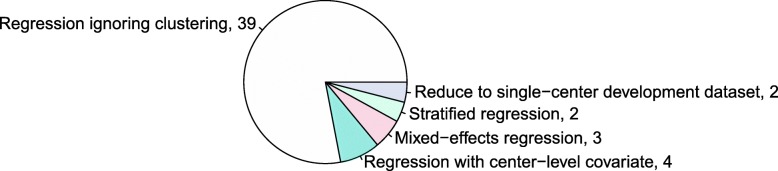
Analysis techniques used for model building in 50 randomly selected multicenter studies

The vast majority of studies (39/50) completely ignored the clustered data structure that is typical for multicenter studies during analysis (Fig. 4; 24 used logistic regression models, 13 Cox models, 2 Fine and Gray models). The other studies took clustering into account when building the prediction model in a variety of ways: standard Cox or logistic regression with a center-level covariate (e.g., a fixed center effect, a region effect, a physician experience effect), mixed effects logistic regression, or stratified regression. Two studies split their dataset into a single-center model development set and used the other centers for geographical validation.

**Fig. 5:**
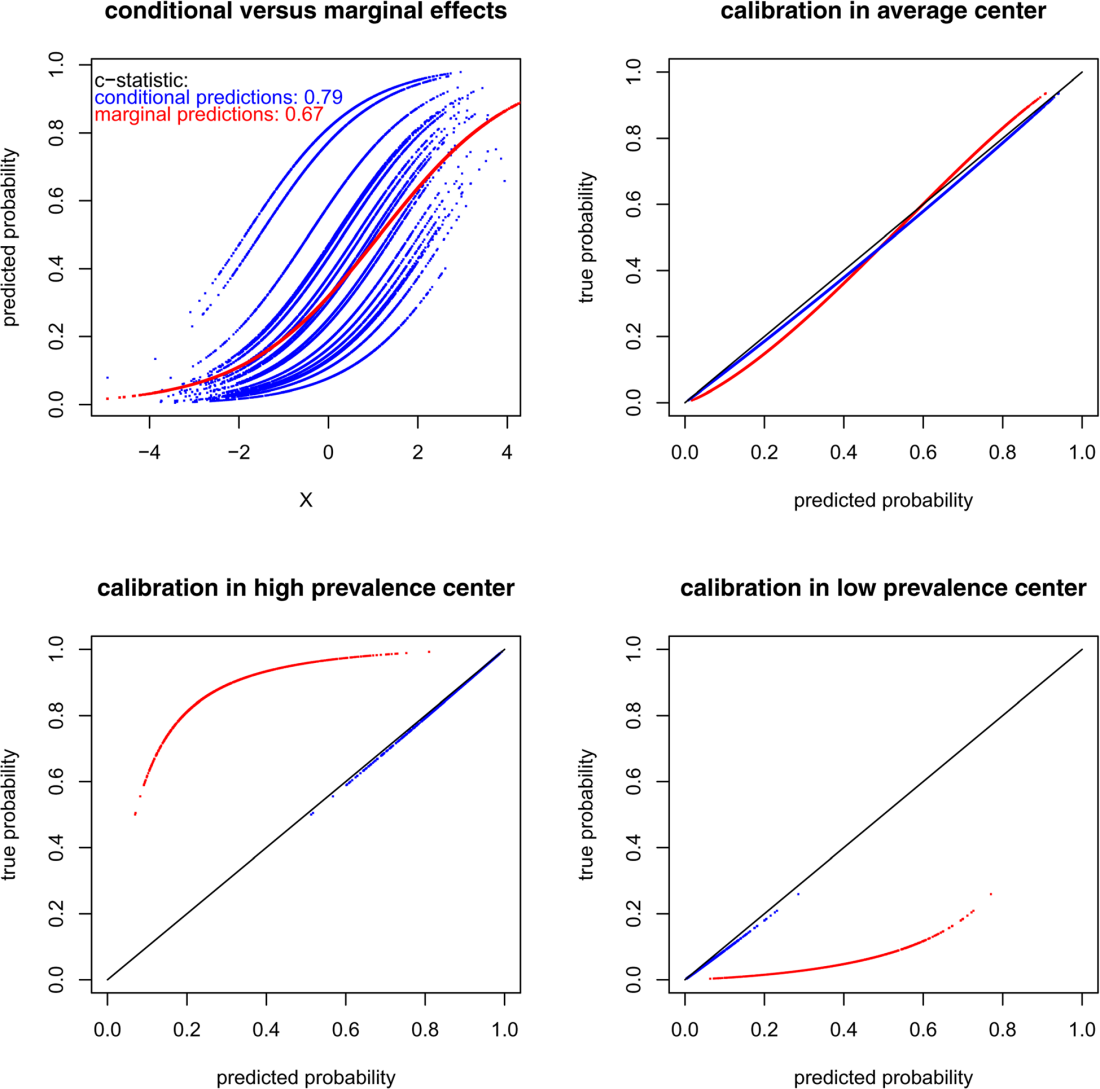
Didactical example of predictions made ignoring center effects, when differences between centers are very large

### Validation of the model

In leave-center out-cross-validation, each cluster is left out of the dataset as a validation set once, while the model is fitted on all other clusters [9]. Only one study used this technique, an IPD-MA that used leave-one-study-out cross-validation.

Ten studies included an external validation in the model development study, two of which used two separate external validation datasets. In 3/10 studies, validation was merely temporal. In 6/10 studies, validation was a mixture of temporal and geographic validation (in a different center or community, four studies used validation data from a different country). Validation in data collected at the same time as model development, but from another center, occurred in 1/10. All validations took place in hospitals or care settings similar to the ones in which the model was developed.

### Multicenter data does not guarantee generalizability

To evaluate in what type of settings a prediction model could be applied, it is crucial that researchers describe the settings from which study data was obtained. In our sample, 23/50 studies failed to do so.

Among the studies that did report on the settings from which data was obtained, 16/27 were single-setting studies: 13 collected all data in tertiary/academic centers, 2 collected all data in community hospitals, and 1 collected all data in veteran affair hospitals. Data from multiple settings (8/27; e.g., a mix of academic and community hospitals, a mix of teaching and non-teaching hospitals) and use of population or community cohort data (3/27) were rare.

Most studies used data from only one country; 11/50 were international studies, and 3/50 did not report where data was collected.

Only 17/50 of studies included a direct or indirect statement on the generalizability of their findings to the target population or the potential applicability in other centers. Eleven studies addressed potential selection bias (including referral bias and spectrum bias) in their study. Five studies critically reflected on generalizability in light of a high disease prevalence in their study population. One study questioned generalizability due to the use of specific devices in the participating centers. More than half the studies (27/50) did not discuss representativeness of the study sample or selection bias, but simply stated that external validation is needed. A few studies (4/50) discussed neither generalizability nor external validity. Only two studies (2/50) claimed generalizability of their model to the target population. The first did so on the basis of using data from a large, multinational registry (*N* = 63,118; 126 centers) covering a heterogeneous population, accrued using consecutive enrolment with a limit on the number of inclusions per month per site to ensure representativeness, with standardized definitions, quality control efforts, and audits. The second (*N* = 1048, 288 centers, a mix of teaching and non-teaching hospitals) did so based on their use of registry data rather than clinical trial data, a claimed lack of selection, recall, and respondent bias, and successful external validation in an independent registry with a lower proportion of severely ill patients than the development dataset.

**Fig. 6:**
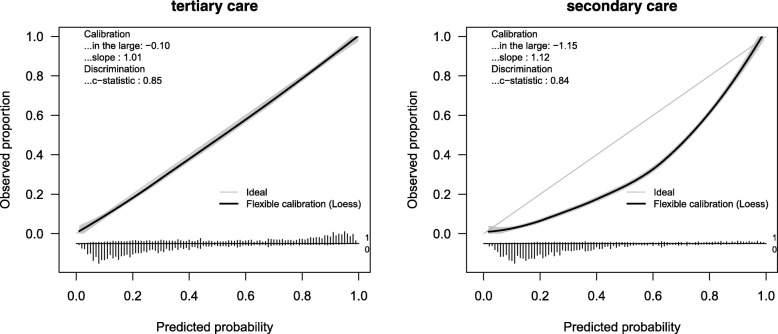
Example of a prediction model built in tertiary care that is generalizable to a new tertiary care center but not transportable to a new secondary care center

## Discussion

This review of the Tufts PACE Clinical Prediction Model Registry indicated that 64% of clinical prediction models published after 2000 were derived from multicenter data. Our survey of published studies resulted in a number of important observations.

Firstly, “multicenter” is a broad term, covering small-scale studies conducted at only two collaborating centers as well as very large international registries. Secondly, all studies had in common that the collected patient data cluster within centers, yet this data structure is most often ignored during analysis. In the minority of studies that did acknowledge the data structure, a broad array of analysis techniques were used, indicating there is no commonly accepted way to build prediction models in clustered data. Thirdly, even though multicenter studies hold the promise of better generalizability to the clinical target population than single-center studies, reporting on the potential generalizability and external validity is not transparent. Nearly one in two studies failed to describe the care settings from which data was obtained, and nearly two thirds of studies failed to critically reflect on the potential generalizability of the developed model to new centers or resorted to vague statements that further validation was needed.

Our findings are in line with Kahan’s, who reviewed multicenter randomized trials and found that the reporting of key aspects was poor and only 29% of studies adjusted for center in the analysis [77]. They are also in line with numerous reviews that signaled incomplete reporting and poor design and analysis for prediction models for diabetes [78], kidney disease [79, 80], cancer [81], neurological disorders [82, 83], and fetal and maternal outcomes in obstetrics [84]. Some have criticized vagueness of reported selection criteria, such that it remained unclear whether participants were selected in an unbiased way [85], and specialist fields contributing the majority of models, which are unlikely to be generalizable to general hospital populations [80]. A common grievance is the paucity of external validations [79–85]. The proportion of multicenter studies was rarely reported in published reviews but seems to vary by specialty [80, 82, 83]. Researchers in neurology have identified the common use of single-center data as one of the main causes for the lack of generalizability of models in their fields [82, 83].

An obvious limitation of our review is that it is limited to 50 risk prediction studies in cardiovascular disease. We expect that in other fields, multicenter studies also differ widely in terms of numbers of included centers and analyses techniques used. A second limitation is that publication bias may have influenced the results. Large-scale studies with many participating centers may have had a higher likelihood of being published, potentially leading to overestimated study sizes and numbers of participating centers in this review.

### Recommendations for research practice

The strengths of a multicenter design are the ability to speed up data collection and the coverage of a broader population. However, the successful conduct of prospective multicenter studies requires careful study organization and coordination, motivated study staff at the participating centers, and a dedicated and experienced method center [86]. Based on our findings, we can make some suggestions to improve the analysis and reporting of multicenter studies.

#### Model development

We recommend using appropriate statistical techniques to analyze clustered data. Studies have shown that standard logistic regression yields suboptimal results when data is clustered [3–5]. First, mixed and fixed effects regression provide valuable insights into the differences in event rate between centers, after adjustment for the patient-level predictors [6]. Second, it is well known that ignoring clustering yields incorrect standard errors (often underestimated). This also affects stepwise regression, which is known to induce “testimation bias” even in unclustered data [87], but is still common. Third, standard regression methods yield regression coefficients and predicted probabilities with a marginal interpretation, averaged in the population, ignoring centers [74, 75]. As illustrated here and elsewhere [4, 76], this has a negative impact on model calibration. Miscalibration gets worse as the differences between centers increase. In contrast, fixed (with center dummies) and mixed effects regression methods yield correct standard errors for patient-level predictors and better calibrated predictions to support decision-making at the center level, even if average center effects are used in new centers [3, 4]. Fourth, modeling center effects also improves discrimination between two patients from different centers [4, 88].

During model building, center-level covariates can be used to tailor predictions to centers with specific characteristics. This captures the combined effects of omitted predictors that vary by center and are not fully captured by the patient-specific covariates (e.g., regional differences in population health, different patient spectra due to referral mechanisms). Those may be extremely difficult to capture otherwise. For example, one may incorporate a predictor in the model that specifies the center type or specialty (e.g., tertiary centers versus others) [8, 89, 90]. Note, however, that standard regression techniques that ignore clustering yield standard error estimates for effects of center characteristics that are typically too small.

When using fixed or mixed effects regression, one may check for center-predictor interaction (or random slopes). This type of interaction may be rare and have little influence on predictions [91].

Fixed and mixed effects regression models can easily be fitted with commonly used statistical software. To apply such models in a new center, an average intercept can be used (which is a random intercept of zero in a mixed effects model; in a fixed effects model, this is not straightforward) and simple updating techniques can yield predictions tailored to new centers [13, 92].

#### Model validation

A geographical split into a single-center development dataset and a validation set consisting of data from the other centers may alleviate the need to address clustering during model building but is very inefficient as only a part of the available data is used for developing the model.

In contrast, leave-center-out cross-validation (also known as internal-external cross-validation) makes efficient use of all available data and offers an excellent opportunity to test the generalizability of the model to new centers [9]. To summarize the predictive performance in each center, a simple average performance [9] or an optimism-corrected performance measure can be computed [93]. At the very least, however, the difference in performance between centers should be inspected [94]. Meta-analytic techniques summarize performance and simultaneously quantify between-center heterogeneity, by distinguishing between sampling variability within centers and variance in predictive performance between centers [18, 95]. Note that leave-center-out cross-validation does not resolve data clustering in the development sets and does not guarantee transportability to distinct care settings or populations not represented in the multicenter dataset.

#### Reporting

We recommend identifying the patients and care settings for which the model is intended and transparently reporting the threats to generalizability to this target population. This includes careful reporting of center-level characteristics such as types of centers (e.g., secondary versus tertiary care) and the sample sizes per center. Describing the limitations of the study, including non-representativeness of study samples, is already a key element on the TRIPOD checklist [1]. The STROBE checklist includes the requirement to address potential sources of bias and states that the direction and magnitude of any potential biases should be addressed [96]. We fully endorse these recommendations. Selection bias is of special interest in prediction model studies. The study sample may not be representative of the clinical population in which the developed model is intended to be applied, for example, because data was collected in teaching hospitals by physicians with a research interest in the disease under study (referral bias). The performance of a prediction model is influenced by the case-mix and the disease prevalence [15, 97, 98]. Hence, if specialized centers collected the study data, selection bias may lead to overestimated disease probabilities (miscalibration-in-the-large) or lower specificity. Note that in half of the studies reporting information on study settings, all participating centers were tertiary or academic centers. Arguably, prediction models and decision-support systems are most useful at lower levels of care (secondary and primary), where the patient case-mix is broad, and practitioners need to triage and refer patients efficiently.

#### Design for generalizability

Lastly, multicenter studies have most potential if generalizability is not an afterthought but considered at study design. Frequently, researchers develop prediction models from databases collected for other purposes. However, if prediction is considered in the design phase, centers from diverse settings can participate to ensure the dataset is representative of the intended target population. Moreover, researchers can aim to collect consecutive patients from each participating center, whilst maintaining a good balance in center sample sizes.

## Conclusion

Multicenter designs are very common in prediction model development. Although multicenter studies may provide better insight in the generalizability of developed prediction models, this potential often remains untapped due to the use of unsuitable analysis methods and lack of transparent reporting.

Box 1 An illustration of the consequences of ignoring clustering Standard regression models ignore any differences between centers and have been illustrated to yield worse predictions than mixed or fixed effects regression models in real clinical examples and simulated data [3–5]. Consider the didactical illustration in Fig. 5, where the data is strongly clustered and standard and mixed effects logistic regression models with a single predictor are estimated in a large dataset (*n* = 500,000, simulation details in Appendix) Additional file 1. If standard logistic regression is used (red dots), the large differences in baseline event rates between centers (blue dots for 20 randomly selected centers) go unnoticed. Ignoring this source of variance in the dataset often leads to underestimated standard errors (in this example with 500,000 observations, 0.0034 versus 0.0038). When acknowledging the differences in event rates between centers, a better discrimination can be obtained by using the conditional probabilities (C-statistics 0.79 vs. 0.67). Standard regression methods yield regression coefficients and predicted probabilities with a marginal interpretation, averaged in the population, ignoring centers. The effect estimates are typically closer to zero, than when clustering is taken into account [74, 75]. Hence, in Fig. 5, the slope of the marginal predictions (red dots) is less steep than that of the mixed effect model’s conditional predictions in each center (blue dots). This has implications for model calibration, as described in detail elsewhere [4, 76] and illustrated in the second panel. The predictions from the marginal model are too moderate for patients in the average center. Because differences in event rate between centers are ignored by the standard logistic regression model, predicted probabilities will often be systematically over- or underestimated in individual centers, in contrast to the mixed effects model (third and fourth panel). One may argue that calibration within centers is most important in the context of prediction modeling, because test results are interpreted and clinical decisions are made in the individual centers. A second example, with small but realistic differences between centers, is provided in Appendix.

Box 2 Illustrations of non-transportability When a model is developed from a selected high-risk population, it may not generalize well to a general hospital population or a lower level of care. The Essen Stroke Risk Score to predict recurrent cardiovascular events was developed from cerebrovascular trial patients, a selective and high-risk population. When validated in a registry representative of stable outpatients, it yielded consistently overestimated cardiovascular event rates in each risk stratum, except the lowest [22].

As another example, consider a prediction model for ovarian cancer that was developed in a multicenter dataset of 2263 patients in 10 tertiary care centers (ratio between the largest and the smallest center sample size 4:1), which is a typical example of a multicenter dataset judging by the results of the review. In tertiary care, the average patient age was 49 (sd 16) and the average log-transformed CA125 value was 4.05 (sd 1.78). The outcome prevalence was 0.43. The linear predictor of the prediction model was − 5.79 + 0.04 × age + 0.83 × log(CA125). In Fig. 6, the calibration plot shows that this model is generalizable to an independent tertiary care center.

In secondary care, patients were slightly younger (average 47, sd 16) and had lower log-transformed CA125 values (average 3.45, sd 1.32). The true outcome prevalence was 0.17. The model developed in data from tertiary care was not transportable to secondary care. The predicted probabilities of experiencing the event were severely overestimated (expected number of events 3126, observed number of events 1629, calibration plot in Fig. 6). Transportability to settings not represented in the development data cannot be assumed, regardless of the statistical modeling used.

The overestimation of the event probability may be solved by re-estimating the prediction model’s intercept in secondary care [13]. However, it is interesting to note that the effects of age and the CA125 value were slightly smaller in tertiary care than in secondary care (see Appendix). This may occur in real life, due to referral bias. Specialized care may see a larger share of puzzling or unusual cases (e.g., young diseased people).

### Additional files

Additional file 1: Simulation code. (PDF 204 kb)

## Figures and Tables

**Fig. 7:**
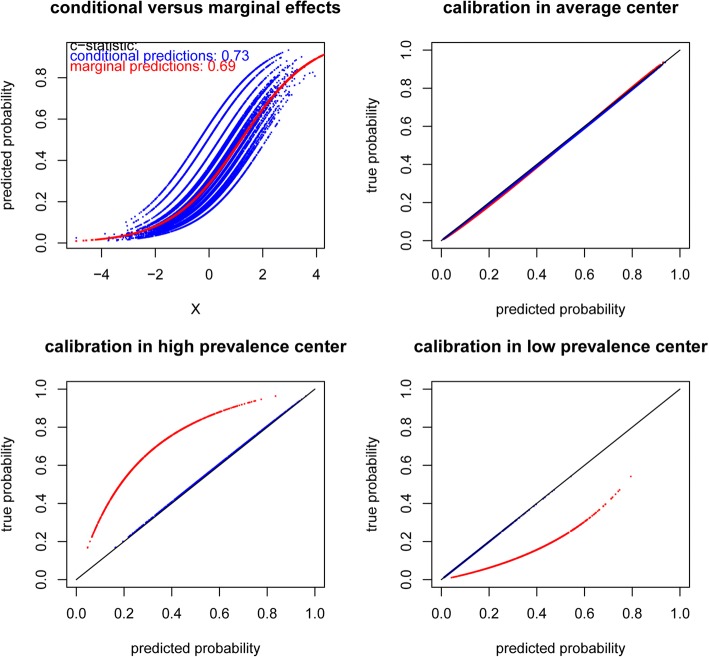
Didactical example of predictions made ignoring center effects, when differences between centers are small

## Appendix (Fig. 7)

### Simulation details

#### Illustration 1 Ignoring clustering

We generated a dataset of 500,000 patients. A single standard normal predictor **X** (which may be thought of as a linear combination of multiple predictors) had a true effect of 0.8. The true intercept was − 1. Random effects (**ri**) of 500 centers (1000 patients each) were generated with a mean of zero and a standard deviation of 1 or 0.5 (two scenarios). The true disease probability was calculated by applying the inverse logit transformation to − 1 + **ri** + 0.8 **X**. By comparing the disease probability to a random draw from a uniform distribution on the interval [0,1], the binary outcome vector **Y** was created. The event rate is 0.33 in scenario 1 and 0.31 in scenario 2.

In the generated datasets, a standard and mixed effects (random intercept) logistic regression model were fitted. We plotted the predicted probabilities of each model as a function of *X*. We calculated the C-statistic of both. In addition, we plot the predicted probabilities against the true probabilities in an average-prevalence center, a high-prevalence center, and a low-prevalence center. The R code is provided online.

The results of scenario 1, which has extreme clustering, are shown in the main manuscript for didactical reasons. The plot below shows the results of scenario 2, with small between-center differences, akin to what may be found in a study using data from various similar centers (e.g., all tertiary centers).

#### Illustration 2 Non-transportability

The estimated random intercept logistic regression models in a clinical database (3439 patients in 12 oncology referral centers and 1664 patients in 8 general hospitals) were taken to be the true models in this example. In tertiary care, this is log(*p*(*Y* = 1)/(*p*(*Y* = 0)) = − 5.77 + 0.04 × age + 0.85 × log(biomarker). The proportion of residual variance in *Y* attributable to differences between centers is 0.06. In secondary care, this is log(*p*(*Y* = 1)/(*p*(*Y* = 0)) = − 7.77 + 0.05 × age + 0.91 × log(biomarker). The proportion of residual variance in *Y* attributable to differences between centers is < 0.01.

A dataset to develop a clinical prediction model was constructed by sampling age and biomarker values from the tertiary care dataset. This way, we assure a realistic joint distribution of predictor values. The properties of the multicenter model development dataset were informed by findings of the review. We first sampled 10 centers without replacement, and within each center, we sampled patients such that the total sample size was 2263 and the ratio between the largest and the smallest center sample size was 4 to 1. After predictor values were sampled, *Y* values were generated according to the true model in tertiary care, using center-specific intercepts.

In the created development dataset, a random intercept logistic regression model was fitted to generate the prediction model.

Next, a simple random sample of 10,000 patients was taken with replacement from the tertiary care database, again generating *Y* values according to the sampled predictor values and the true model coefficients, but this time assuming an average center intercept. By creating such a large validation set (“augmenting” or “upstrapping” the real tertiary care database), we can trust that observed differences in predictive performance in this illustration are not due to random sampling error. Generating a 0/1 outcome based on the model-based true probability for a certain vector of predictor values (e.g., age 49 and log-biomarker value 4) assures that the event is observed with a certain probability each time this predictor value combination is sampled,and avoids exact replicates of observations and deterministic relations in the dataset. The generated dataset is the validation set to assess discrimination and calibration in tertiary care.

Another simple random sample of 10,000 patients was taken with replacement from the secondary care database, while *Y* values were generated according to the true model coefficients in secondary care. This is the validation set to assess discrimination and calibration in secondary care.

## Abbreviations

CPM: Clinical predictive model
IPD-MA: Individual patient data meta-analysis
LR: Logistic regression
