# Supplementary material for: Untapped potential of multicenter studies: a review of cardiovascular risk prediction models revealed inappropriate analyses and wide variation in reporting
Source: Diagn Progn Res. 2019 Feb 22;3:6. doi: 10.1186/s41512-019-0046-9 (PMC6460661; doi:10.1186/s41512-019-0046-9)
Supplement: Supplementary file 1 — Simulation code. (PDF 204 kb) [file 41512_2019_46_MOESM1_ESM.pdf]

Simulation R- code

```
int<--1 #True intercept
re<-rnorm(500, 0, 0.5) #True random effects for 500 centers, with standard deviation 0.5
res<-rep(re, each=1000) #Random effects in a vector, 1000 patients in each center
x<-rnorm(500*1000, 0, 1) #Standard normal predictor

logit<- int + 0.8 * x + res #Generate the true outcome
p<-plogis(logit) #True probalities
y<-ifelse(runif(500*1000)<=p, 1, 0) #True binary outcome

marg.model<-glm(y~x, family="binomial") #Fit the standard logistic regression model
logit.m<- marg.model$coefficients[1] + marg.model$coefficients[2]*x
p.m<-plogis(logit.m) #Marginal predictions

library(lme4)
cond.model<-glmer(y~x+(1|as.factor(res)), family="binomial") #Fit the mixed effects logistic regression model
logit.c<-predict(cond.model, newdata=data.frame(x,as.factor(res)))
p.c<-plogis(logit.c) #Conditional predictions

library(pROC)
auc(y, p.m) #The c-statistics
auc(y, p.c)

plot(x[1:20000],p.c[1:20000], xlab="X", ylab="predicted probability", main="conditional versus marginal effects", pch="." , col="blue") #plot conditional predictions for first 20 centers
points(x,p.m, col="red",pch=".", cex=2) #plot marginal predictions
p.average<-plogis(int+0.8*x) #true probability in average center
p.high<-plogis(int+0.8*x+max(res)) #true probability in center with highest random intercept
p.low<-plogis(int+0.8*x+min(res)) #true probability in center with lowest random intercept
p.c.a<-plogis(fixef(cond.model)[1]+x*fixef(cond.model)[2]) #prediction in the average center (random intercept zero)

plot(p.m, p.average,pch=".", main="calibration in average center", xlim=c(0,1), ylim=c(0,1), xlab="predicted probability", ylab="true probability", col="red") # Calibration in average center for marginal prediction
points(p.c.a, p.average, pch=".", col="blue") # Calibration in average center for conditional prediction

plot(p.m[res==max(res)], p.high[res==max(res)],pch=".", main="calibration in high prevalence center", xlim=c(0,1), ylim=c(0,1), xlab="predicted probability", ylab="true probability", col="red") #Calibration in high-prevalence center for marginal prediction
points(p.c[res==max(res)],p.high[res==max(res)],pch=".", col="blue" ) #Calibration in high-prevalence center for conditional prediction

plot(p.m[res==min(res)], p.low[res==min(res)],col="red", pch=".", main="calibration in low prevalence center", xlim=c(0,1), ylim=c(0,1), xlab="predicted probability", ylab="true probability") # Calibration in low-prevalence center for marginal prediction
points(p.c[res==min(res)],p.low[res==min(res)] ,pch=".", col="blue") #Calibration in low-prevalence center for marginal prediction
```
